# Functional differences between the arteries perfusing gas exchange and nutritional membranes in the late chicken embryo

**DOI:** 10.1007/s00360-015-0917-5

**Published:** 2015-06-29

**Authors:** Riazudin Mohammed, Giacomo Cavallaro, Carolina GA Kessels, Eduardo Villamor

**Affiliations:** Department of Pediatrics, Maastricht University Medical Center (MUMC+), Research Institute Growth and Development (GROW) and Cardiovascular Research Institute Maastricht (CARIM), University of Maastricht, P. Debyelaan 25, P.O. Box 5800, 6202 AZ Maastricht, The Netherlands; Neonatal Intensive Care Unit, Department of Clinical Sciences and Community Health, Fondazione IRCCS Cà Granda Ospedale Maggiore Policlinico, Università degli Studi di Milano, Milan, Italy

**Keywords:** Chicken embryo, Fetal circulation, Umbilical, Endothelium, Adrenergic

## Abstract

The chicken extraembryonic arterial system comprises the allantoic arteries, which irrigate the gas exchange organ (the chorioallantoic membrane, CAM) and the yolk sac (YS) artery, which irrigates the nutritional organ (the YS membrane). We compared, using wire myography, the reactivity of allantoic and YS arteries from 19-day chicken embryos (total incubation 21 days). The contractions induced by KCl, the adrenergic agonists norepinephrine (NE, nonselective), phenylephrine (α_1_), and oxymetazoline (α_2_), electric field stimulation (EFS), serotonin, U46619 (TP receptor agonist), and endothelin (ET)-1 and the relaxations induced by acetylcholine (ACh), sodium nitroprusside (SNP, NO donor), forskolin (adenylate cyclase activator), and isoproterenol (β-adrenergic agonist) were investigated. Extraembryonic allantoic arteries did not show α-adrenergic-mediated contraction (either elicited by exogenous agonists or EFS) or ACh-induced (endothelium-dependent) relaxation, whereas these responses were present in YS arteries. Interestingly, the intraembryonic segment of the allantoic artery showed EFS- and α-adrenergic-induced contraction and ACh-mediated relaxation. Moreover, glyoxylic acid staining showed the presence of catecholamine-containing nerves in the YS and the intraembryonic allantoic artery, but not in the extraembryonic allantoic artery. Isoproterenol- and forskolin-induced relaxation and ET-1-induced contraction were higher in YS than in allantoic arteries, whereas serotonin- and U46619-induced contraction and SNP-induced relaxation did not significantly differ between the two arteries. In conclusion, our study demonstrates a different pattern of reactivity in the arteries perfusing the gas exchange and the nutritional membranes of the chicken embryo.

## Introduction

In amniotes, there are four fetal membranes: the amnion that surrounds the fetus and protects it from mechanical and physiological stress; the yolk sac (YS) that in birds and some reptiles surrounds the egg yolk; the allantois, which is an extension of the embryonic bladder and stores excretory products; and the chorion, which is formed by extraembryonic mesoderm and fuses with either the YS to form the choriovitelline placenta or with the allantois to form the chorioallantoic placenta (Renfree et al. 2013). Most mammals rely on both types of placenta at least for some periods of pregnancy, and even in humans the YS is crucial for the survival of the early embryo (Jones and Jauniaux 1995; Renfree et al. 2013). Since the placenta is the organ that transports nutrients, respiratory gases, and wastes between the maternal and fetal systems, avian embryos do not have—sensu stricto—placenta. However, avian embryos are endowed with a gas exchange organ, the chorioallantoic membrane (CAM), and a choriovitelline nutritional organ, the YS membrane (Speake et al. 1998; van Golde et al. 1996; White 1974; Dzialowski et al. 2011). Interestingly, these two extraembryonic membranes are perfused by two separate arterial systems.

**Fig. 1:**
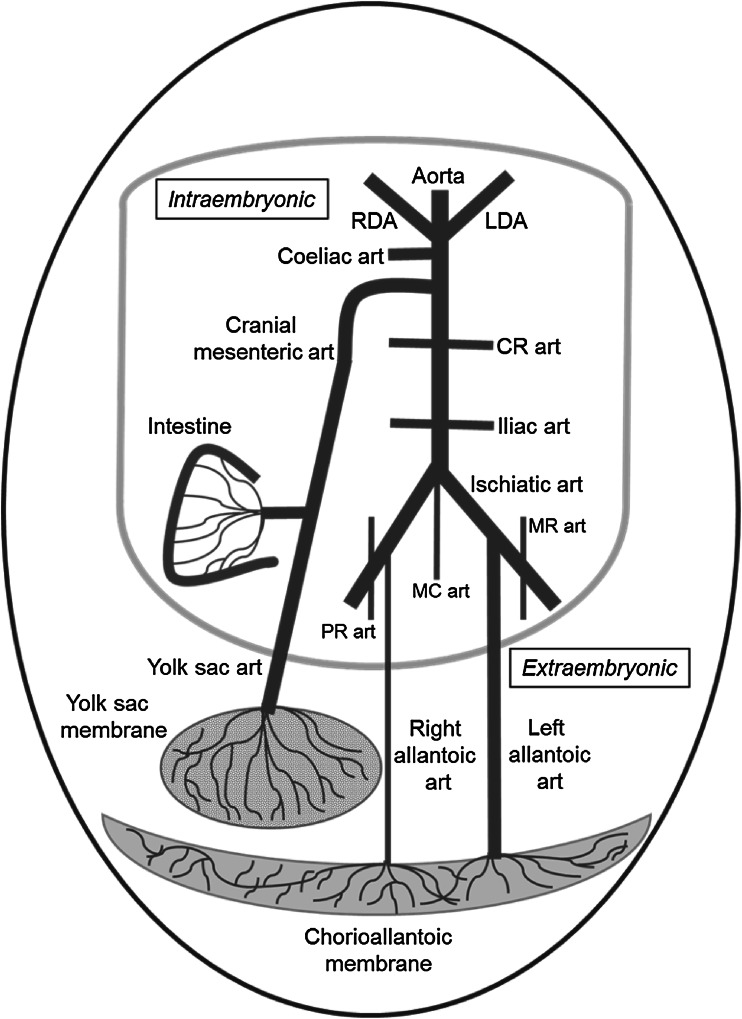
Schematic representation of the origin of the major abdominal arteries and the umbilical circulation of the chicken embryo. *Art* artery; *CR* cranial renal; *LDA* left ductus arteriosus; *MC* median caudal; *MR* middle renal; *PR* posterior renal; *RDA* right ductus arteriosus

The CAM is supplied by two arteries (from now named allantoic arteries) derived from the right and left ischiatic arteries (Levinsohn et al. 1984) (Fig. 1). The allantoic arteries bring the deoxygenated blood into contact with CAM, where exchange of gases through the shell occurs, and the allantoic vein returns oxygenated blood to embryo (van Golde et al. 1996; White 1974; Dzialowski et al. 2011). The YS provides the chicken embryo with essential nutrients for its growth. Protein, fat, carbohydrates, and minerals are stored in the yolk of the hen egg for future utilization by the embryo (Yadgary et al. 2013; Speake et al. 1998). The utilization of these nutrients is enabled by the YS membrane. During the first week of incubation, the inner endodermal layer of the YS membrane increases in area and spreads over the surface of the yolk, concomitant with the vascularization of the membrane by the outer supportive mesodermal layer. By embryonic day 10, the entire yolk is surrounded and vascularized, and the endodermal cells of the YS membrane have developed into columnar epithelial absorptive cells (Speake et al. 1998). The late embryo YS membrane is supplied by a branch (from now named YS artery) of the cranial mesenteric artery (Levinsohn et al. 1984; White 1974) (Fig. 1).

In the last few years, the chicken embryo has emerged as a suitable model for studying developmental vascular biology. The reactivity of chicken embryo systemic and pulmonary vessels has been profusely studied (Agren et al. 2007, 2008, 2009; Dzialowski et al. 2011; le Noble et al. 2000; Moonen et al. 2012; Moonen and Villamor 2011; Rouwet et al. 2000; Schuurman and Villamor; 2010; Villamor et al. 2002; Cogolludo et al. 2009; Flinsenberg et al. 2010; Ruijtenbeek et al. 2002; van der Sterren et al. 2011; Villamor et al. 2004; Zoer et al. 2010a, b). However, little is known about the extraembryonic vessels of the chicken embryo. Lindgren et al. characterized the reactivity of the small arteries of the CAM (Lindgren et al. 2010, 2011) but the reactivity of chicken umbilical arteries has not been yet investigated. In the present study, we hypothesized that the separation of the respiratory and nutritional “placental” functions in the chicken embryo would be accompanied by a concomitant functional specialization of the arteries supplying the CAM and the YS membrane. We tested our hypothesis by analyzing the responsiveness of isolated allantoic and YS arteries to several vasoactive agents which play a relevant role in the control of chicken embryo vascular tone.

## Methods

### Incubation of chicken (*Gallus gallus*) embryos and vessel isolation

All experimental procedures were carried out in accordance with the Dutch Law on Animal Experimentation and the European Directive for the Protection of Vertebrate Animals Used for Experimental and Other Scientific Purposes (86/609/EU) and approved by the Committee on Animal Experimentation of the University of Maastricht. Fertilized eggs from White Leghorn chickens (‘t Anker, Ochten, The Netherlands) were incubated at 37.8 °C, 45 % humidity and rotated once per hour over an angle of 90° (Incubator model 25HS, Masalles Comercial, Spain).

Umbilical vessels were sampled at 19 days of incubation (E19, hatching at 21 days). In a limited number of experiments (see “Results”), vessels were sampled at E15. The extent of the egg air space was determined by candling, and the overlying shell was removed. A cut about 2 cm long was then made in the shell membrane and the underlying CAM, the line of incision being chosen so as to avoid major blood vessels. By gripping the embryo’s beak, it was possible to draw it out through the incision in the CAM and lay it on its back in a Petri-dish coated with silicon. The YS was tipped out and the embryo was killed by decapitation. The extraembryonic allantoic vessels were separated from the CAM close to their point of insertion into it. The preparation was rinsed with cold Krebs–Henseleit (KRB) buffer and a midline laparotomy and sternotomy were performed. With the aid of a dissecting microscope, the ischiatic and the cranial mesenteric arteries were located, and the intra- and extraembryonic trajectories of the allantoic and YS arteries were followed. In situ external diameter of the vessels was determined with the aid of an eyepiece scale which was incorporated in the dissecting microscope. Vascular rings were obtained from the ischiatic artery (distal to the origin of the allantoic artery), the proximal intraembryonic part of the allantoic artery (immediately after its origin in the ischiatic artery), the distal intraembryonic part of the allantoic artery (immediately before its exit from the abdominal cavity), the extraembryonic part of the allantoic artery (in the middle part of its trajectory between the embryo and the CAM), and the extraembryonic part of the YS artery.

### Recording of arterial reactivity

Vascular rings were mounted as ring segments between an isometric force transducer and a displacement device in a myograph (model 610 M; Danish Myo Technology, Aarhus, Denmark). The myograph organ bath (5 ml volume) was filled with KRB buffer maintained at 39 °C and bubbled with 5 % O_2_/90 % N_2_/5 % CO_2_ (Po_2_ ~ 7 kPa). In a limited number of experiments (see results), organ baths were bubbled with 21 % O_2_/74 % N_2_/5 % CO_2_ (Po_2_ ~ 18 kPa). After an equilibration period, the vessels were distended to a resting tension corresponding to a transmural pressure of 2.66 kPa. This pressure corresponds to the mean arterial blood pressure reported in 19days chicken and elicits the highest contractile response to KCl, as determined in previous experiments. E15 vessels were distended to a resting tension corresponding to a transmural pressure of 1.33 kPa. After 30 min of incubation at basal tone, a control reference contraction was elicited by raising the K^+^ concentration of the buffer to 62.5 mM. The preparations were washed three times and allowed to recover before a new stimulation.

### Contractile responses

Concentration–response curves to KCl (31.25–125 mM), the nonselective adrenergic receptor agonist norepinephrine (NE, 10 nM–0.1 mM), the α_1_-adrenergic agonist phenylephrine (10 nM–0.1 mM), the α_2_-adrenergic agonist oxymetazoline (10 nM–0.1 mM), serotonin (5-HT, 1 nM–10 μM), the thromboxane/prostaglandin H_2_ (TP) receptor agonist U46619 (10 nM–3 μM), and endothelin (ET)-1 (0.1 nM–0.1 μM) were constructed by increasing the organ chamber concentration of the drug, by cumulative increments after a steady-state response had been reached with each increment. When two or more agonists were studied in the same arterial preparation, the vessels were repeatedly washed and allowed to equilibrate for at least 30 min. If the tone did not recover to resting level, the vessels were discarded from further experiments. Sympathetic neuroeffector mechanisms were studied using electrical field stimulation (EFS, 0.25–16 Hz, 2 ms, 85 mA) via two platinum electrodes that were placed in the axial direction of the blood vessel. Constant-current pulses were delivered by a stimulator (Technical Services, Universiteit Maastricht, Maastricht, The Netherlands).

### Relaxant responses

Concentration–response curves to relaxant agents were constructed following establishment of stable contraction with KCl (62.5 mM) or 5-HT (1 μM). The muscarinic receptor agonist acetylcholine (ACh, 10 nM-0.1 mM), the nitric oxide (NO) donor sodium nitroprusside (SNP, 10 nM–0.1 mM), the β-adrenoceptor agonist isoproterenol (10 nM–0.1 mM), the β_3_-adrenoceptor agonist BRL 37344 (10 nM–10 μM), the adenylate cyclase activator forskolin (10 nM–10 μM), ATP (10 nM–10 μM), and the calcium ionophore A23187 (10 nM–10 μM) were studied. Some experiments were performed in endothelium-denuded vessels (endothelium was removed by gentle rubbing of the vessel lumen with a horsetail hair, as previously described) (Agren et al. 2008; Schuurman and Villamor 2010) or in the presence of the NO synthase inhibitor Nω-Nitro-l-arginine methyl ester (L-NAME, 0.1 mM), or the cyclooxygenase inhibitor indomethacin (10 μM).

### Glyoxylic acid fluorescence histochemistry

To demonstrate the presence of catecholamine-containing nerves, vessels were stained with glyoxylic acid as previously described (Agren et al. 2007). Whole mount preparations of vascular rings were incubated, while in the organ bath, in 2 % glyoxylic acid and 10 % sucrose in phosphate buffer for 30 min at room temperature. After this, the rings were air-dried (30 min.), stretched at 100 °C for 4 min., and enclosed with entellan and a coverslip. Glyoxylic acid-induced fluorescence was visualized with a Nikon E600FN microscope (Nikon, Tokyo, Japan), coupled to a standard Bio-Rad 2100 MP (Bio-Rad, Hemel Hempstead, UK) two-photon system (objective Fluo 20X, laser 800 W, 560 DCLPXR dichromic mirror, HQ 450LP blocking filter, HQ 530SP emission filter).

### Drugs and solutions

The composition of the KRB buffer was (in mM): NaCl 118.5, MgSO_4_ 1.2, KH_2_PO_4_ 1.2, NaHCO_3_ 25.0, KCl 4.7, CaCl 2.5 and glucose 5.5. Solutions containing different concentrations of K^+^ were prepared by replacing part of the NaCl of the KRB buffer by an equimolar amount of KCl. U46619 was obtained from Cayman Chemical (Ann Arbor, MI). The other drugs were from Sigma (St. Louis, MO). All drugs were dissolved initially in distilled deionized water (except U46619, A23187 and BRL 37344 in DMSO and forskolin and indomethacin in ethanol) to prepare adequate stock solutions and further dilutions were also made in deionized water. The final bath concentration of DMSO or ethanol did not exceed 0.1 % and had no effect on mechanical activity.

### Data analysis

Results are shown as mean ± SE of measurements in *n* embryos. Contractions are expressed in terms of active wall tension (mN/mm, calculated as the force divided by twice the length of the segment) or as a percentage of the reference contraction to KCl (62.5 mM) performed for each individual ring at the beginning of the experiment. The relaxant responses are expressed as the percentage of reduction of the contraction induced by KCl or 5-HT. Sensitivity/potency (expressed as pEC_50_ = −logEC_50_) and efficacy (expressed as E_max_) were calculated by nonlinear regression analysis of the concentration–response curves. Differences between mean values were assessed by Student’s *t* test or one-way ANOVA followed by Bonferroni’s post hoc t test. Differences were considered significant at a *p* < 0.05. All analyses were performed using GraphPad Prism (version 5.00 for Windows, GraphPad Software, San Diego California USA, www.graphpad.com).

## Results

### Non-adrenergic contractile responses

**Fig. 2:**
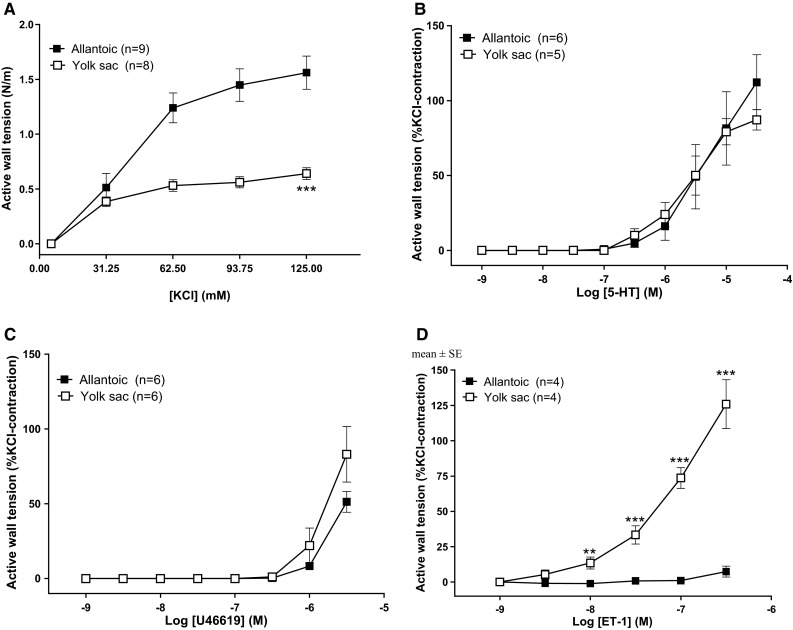
Contractile effects of KCl (**a**), serotonin (5-HT, **b**), the TP receptor agonist U46619 (**c**), and endothelin-l (ET-l, **d**) in extraembryonic arteries (allantoic and yolk sac) from 19-day chicken embryos. Data points are presented as mean ± SE. While the KCl contractions are expressed in mN/mm, the contractions induced by U46619 and ET-I are expressed as percent of the reference KCI-induced contraction in the same vessel. **, ***p < 0.01, 0.001 for difference (yolk sac vs. allantoic) in *E*_max_ or at the individual value (in panel **d**) because *E*_max_ was not reached

Unless otherwise stated, all the results refer to E19 vessels. Isolated extraembryonic allantoic and YS artery rings responded with a tonic contraction to K^+^ (KCl) up to a concentration of 125 mM (Fig. 2a). The response was significantly higher in the allantoic when compared to the YS artery. The effect of all other contractile agents was subsequently expressed as % of the contraction induced by K^+^ (62.5 mM). When the right and left allantoic arteries were compared, the latter showed a higher in situ external diameter (right: 0.52 ± 0.03 mm, *n* = 12; left: 2.11 ± 0.09 mm, *n* = 12; *p* < 0.05) and a higher contractile response to K^+^ (62.5 mM, right: 1.21 ± 0.11 mN/mm, *n* = 12; left: 2.12 ± 0.18 mN/mm, *n* = 12; *p* < 0.05). 5-HT induced a concentration-dependent contraction of extraembryonic allantoic and YS artery rings with similar potency (pEC_50_ allantoic: 5.17 ± 0.17, *n* = 5; pEC_50_ YS: 5.56 ± 0.15, *n* = 5) and efficacy (*E*_max_ allantoic 118.09 ± 10.90; *E*_max_ YS: 87.24 ± 6.77) (Fig. 2b). U46619 (Fig. 2c) and ET-1 (Fig. 2d) induced concentration-dependent contractions with threshold concentrations of 0.3 μM and 10 nM, respectively. Dose–response curves to U46619 and ET-1 could not be adequately fitted because maximum effect was not reached at the highest concentration of the drug used. As shown in Fig. 2d, the contractile effect of ET-1 was significantly higher in the YS arteries than in the allantoic arteries. ET-3 did not induce a significant vasomotor effect in any of the vessels studied (data not shown).

### Adrenergic responsiveness

**Fig. 3:**
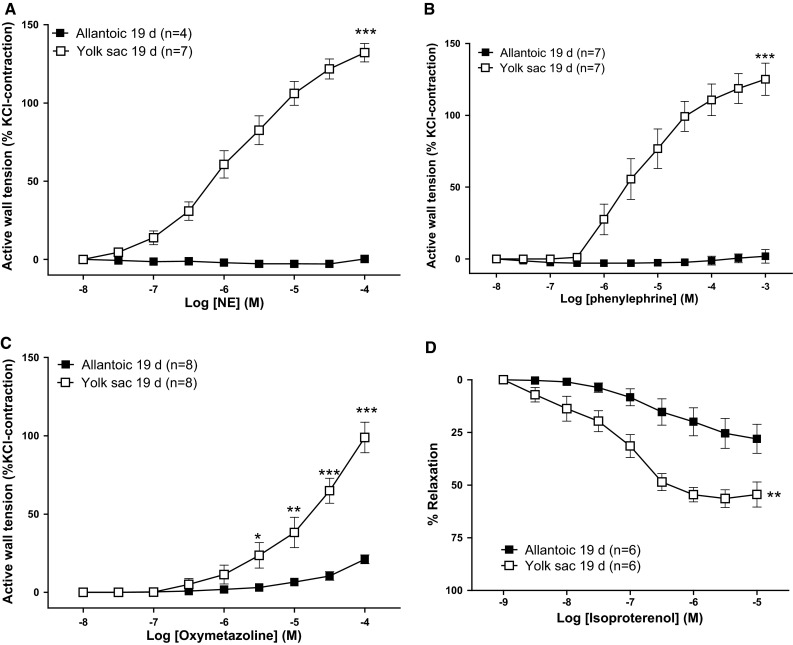
Concentration-dependent effects of adrenergic agonists in extraembryonic arteries (allantoic and yolk sac) from 19-day chicken embryos. Contractile effects of the nonselective adrenergic receptor agonist norepinephrine (NE, **a**), the α_1_-adrenergic agonist phenylephrine (**b**), the α_2_-adrenergic agonist oxymetazoline (**c**), and the β-adrenergic agonist isoproterenol (**d**). For the isoproterenol experiments, vessels were pre-contracted with KCl (62.5 mM). Data points are presented as mean ± SE. *, **, ***p < 0.05, 0.01, 0.001 for difference (yolk sac vs. allantoic) in *E*_max_ or at the individual value (in panel **c**) because *E*_max_ was not reached

The non-selective adrenergic agonist NE, the α_1_-adrenoceptor agonist phenylephrine, and the α_2_-adrenoceptor agonist oxymetazoline all induced concentration-dependent contractions in segments of the YS artery (Fig. 3a–c). In contrast, neither NE nor phenylephrine contracted the extraembryonic allantoic artery (Fig. 3a, b) and oxymetazoline evoked a weak contraction of this vessel when compared with the YS artery (Fig. 3c). The lack of responsiveness of allantoic arteries to NE and phenylephrine persisted when the vessels were bubbled with 21 % O_2_ instead of 5 % (data not shown). The β-adrenoceptor agonist isoproterenol relaxed segments of the YS and the extraembryonic allantoic artery (pre-contracted with 62.5 mM KCl) in a concentration-dependent manner. Isoproterenol showed a higher relaxant efficacy (*p* < 0.05) in the YS (*E*_max_: 57.32 ± 3.69, *n* = 6) than in the extraembryonic allantoic arteries (*E*_max_: 25.32 ± 2.45, *n* = 6). The β_3_-adrenoceptor agonist BRL 37344 did not induce a significant vasomotor effect in any of the vessels studied (data not shown).

**Fig. 4:**
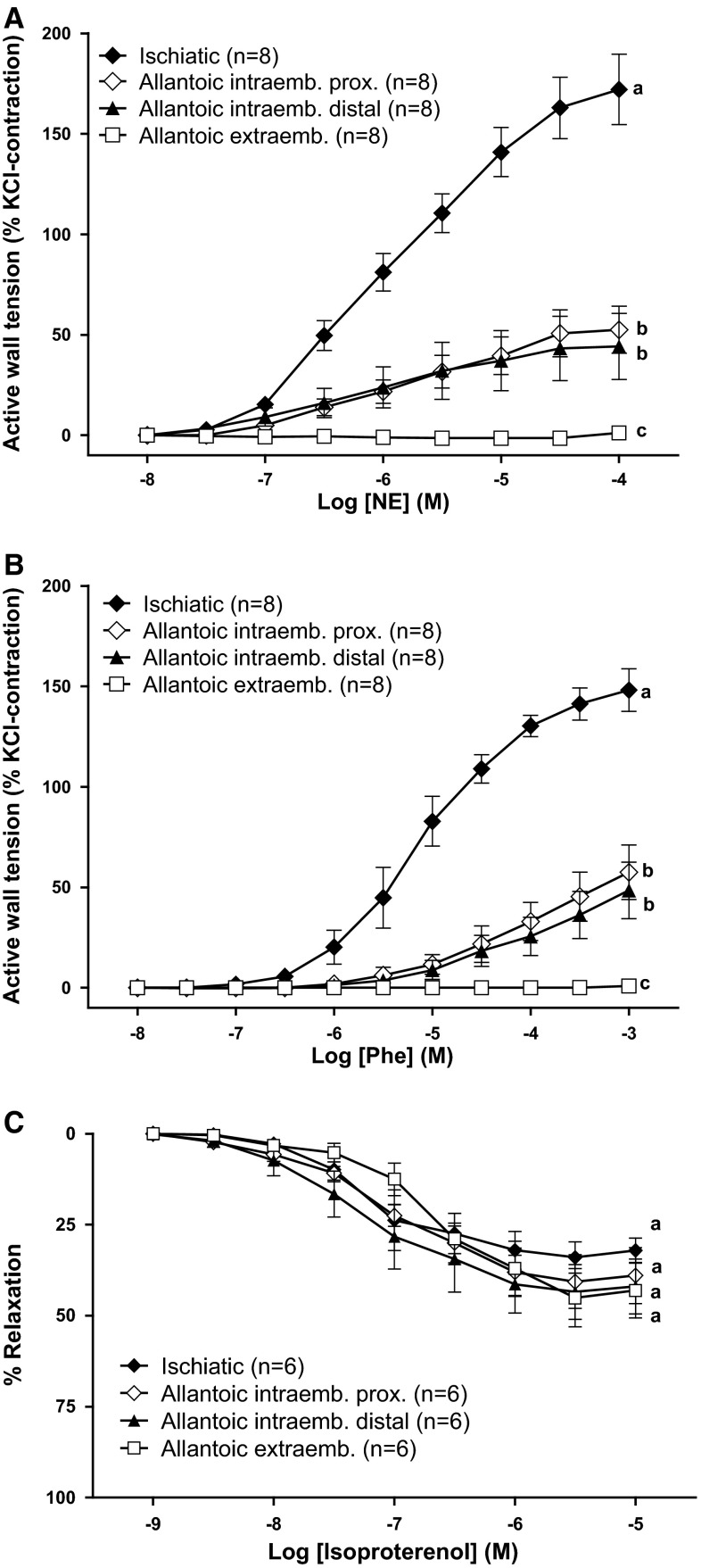
Segmental differences in the responsiveness of the allantoic artery to adrenergic agonists. Contractile effects of the nonselective adrenergic receptor agonist norepinephrine (NE, **a**), the α_1_-adrenergic agonist phenylephrine (Phe, **b**), and the β-adrenergic agonist isoproterenol (**c**) in ischiatic, allantoic intraembryonic (proximal and distal) and allantoic extraembryonic arteries from 19-day chicken embryos. For the isoproterenol experiments, vessels were pre-contracted with KCl (62.5 mM). Data points are presented as mean ± SE. Groups without a *common letter* have a significantly different *E*_max_

To investigate whether the lack of responsiveness to α-adrenoceptor agonists was a specific property of the extraembryonic part of the allantoic artery, we analyzed the response to adrenergic agonists in intraembryonic segments of the artery as well as in rings of the ischiatic artery (the vessel from which the allantoic arteries originate). As shown in Fig. 4a, b, NE and phenylephrine evoked a concentration-dependent contraction of ischiatic and intraembryonic segments of the allantoic artery. Interestingly, a progressive distal decrease in the efficacy of NE and phenylephrine was observed. The relaxation evoked by isoproterenol did not significantly change along the allantoic artery (Fig. 4c).

**Fig. 5:**
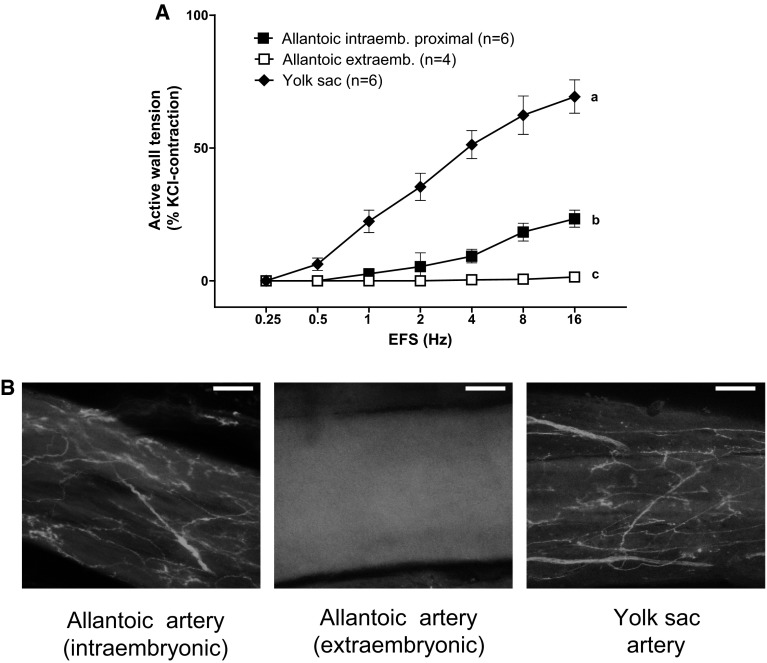
**a** Response to electric field stimulation of extraembryonic arteries (allantoic and yolk sac) and intraembryonic allantoic arteries from 19-day chicken embryos. Data points are presented as mean ± SE. Groups without a *common letter* have a significantly different *E*_max_ (*p* < 0.05). **b** Representative images of glyoxylic acid-induced fluorescence of catecholamines in the allantoic and yolk sac arteries from 19 days chicken embryos. Fluorescent catecholamine-containing nerve fibers were present in the yolk sac artery and the intraembryonic allantoic artery but totally absent in the extraembryonic allantoic artery. Scale bar 100 µm

To investigate the possible role of endogenously released catecholamines in the reactivity of chicken umbilical arteries, rings from the YS artery and the extraembryonic part of the allantoic artery were exposed to EFS. As shown in Fig. 5a, EFS evoked a frequency-dependent contraction in YS arteries but no response in the allantoic arteries. Contraction induced by EFS in the YS artery was abolished by incubation with the nonselective α-adrenergic antagonist phentolamine (1 μM) (data not shown), indicating the involvement of NE release in the vascular response. Finally, we examined the possible presence of catecholamine-containing nerves through glyoxylic acid staining. As shown in Fig. 5b, the YS and the intraembryonic part of the allantoic artery were innervated, whereas perivascular nerves could not be detected in the extraembryonic allantoic artery.

### Relaxation responses

**Fig. 6:**
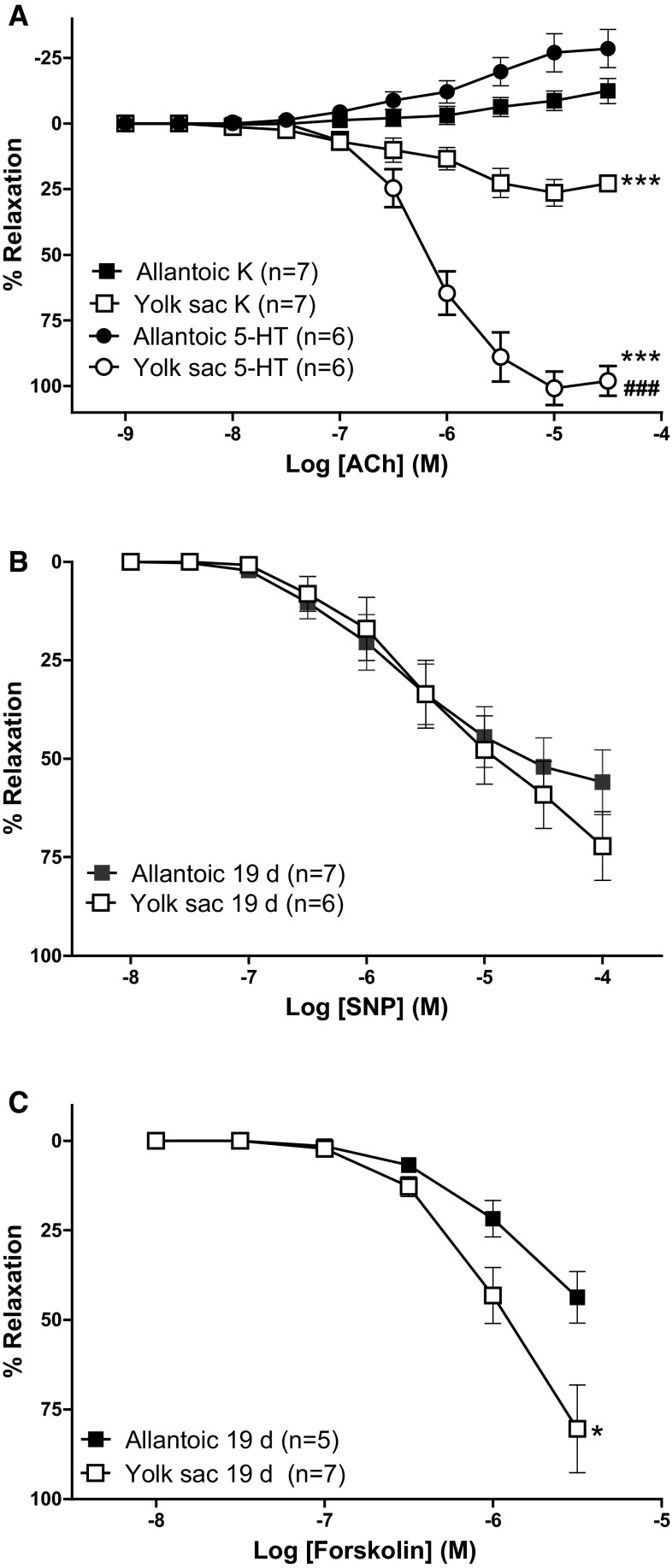
Relaxant effects of acetylcholine (ACh, **a**), sodium nitroprusside (SNP, **b**) and forskolin (**c**) in extraembryonic arteries (allantoic and yolk sac) from 19-day chicken embryos. The vessels were contracted with KCl (62.5 mM) or 5-HT (I!lM, only in panel **a**). Data points are presented as mean ± SE. *, ***p < 0.05, 0.001 (yolk sac vs. allantoic) for difference in *E*_max_ or at the individual value (in panel **c**) because *E*_max_ was not reached.^###^ *p* < 0.001 (5-HT-contracted vs. KCI-contracted) for difference in *E*_max_

The muscarinic agonist ACh relaxed KCl- and 5-HT-contracted rings of the YS artery in a concentration-dependent manner (Fig. 6a). From a concentration >10 μM ACh evoked no further relaxation but contraction in the YS artery rings. The relaxant efficacy (*E*_max_) of ACh was significantly impaired in KCl-contracted YS arteries when compared with 5-HT-contracted vessels. In the extraembryonic allantoic artery rings, either pre-contracted with KCl or 5-HT, no relaxation to ACh was observed (Fig. 6a). From a threshold concentration of 10 μM, extraembryonic allantoic arteries displayed contraction (Fig. 6a). Since in numerous blood vessels ACh-induced relaxation is strongly dependent on oxygen tension, we performed additional experiments, to disclose whether our experimental conditions (i.e., 5 % O_2_) could have been the reason for the lack of responsiveness to ACh. Extraembryonic allantoic arteries bubbled with 21 % O_2_ also failed to show ACh-induced relaxation (data not shown).

As shown in Fig. 6b, c, the NO donor SNP (10 nM–0.1 mM) and the adenylate cyclase activator forskolin (10 nM–0.3 μM) relaxed KCl-contracted YS and allantoic artery rings in a concentration-dependent manner. A maximal effect was not reached for the highest concentration of SNP and forskolin tested. At a concentration of 0.3 μM, the relaxation evoked by forskolin was significantly higher in the YS artery when compared with the extraembryonic allantoic artery (Fig. 6c). Neither ATP nor the calcium ionophore A23187 induced any significant vasomotor response in KCl-contracted YS or allantoic arteries (data not shown).

**Fig. 7:**
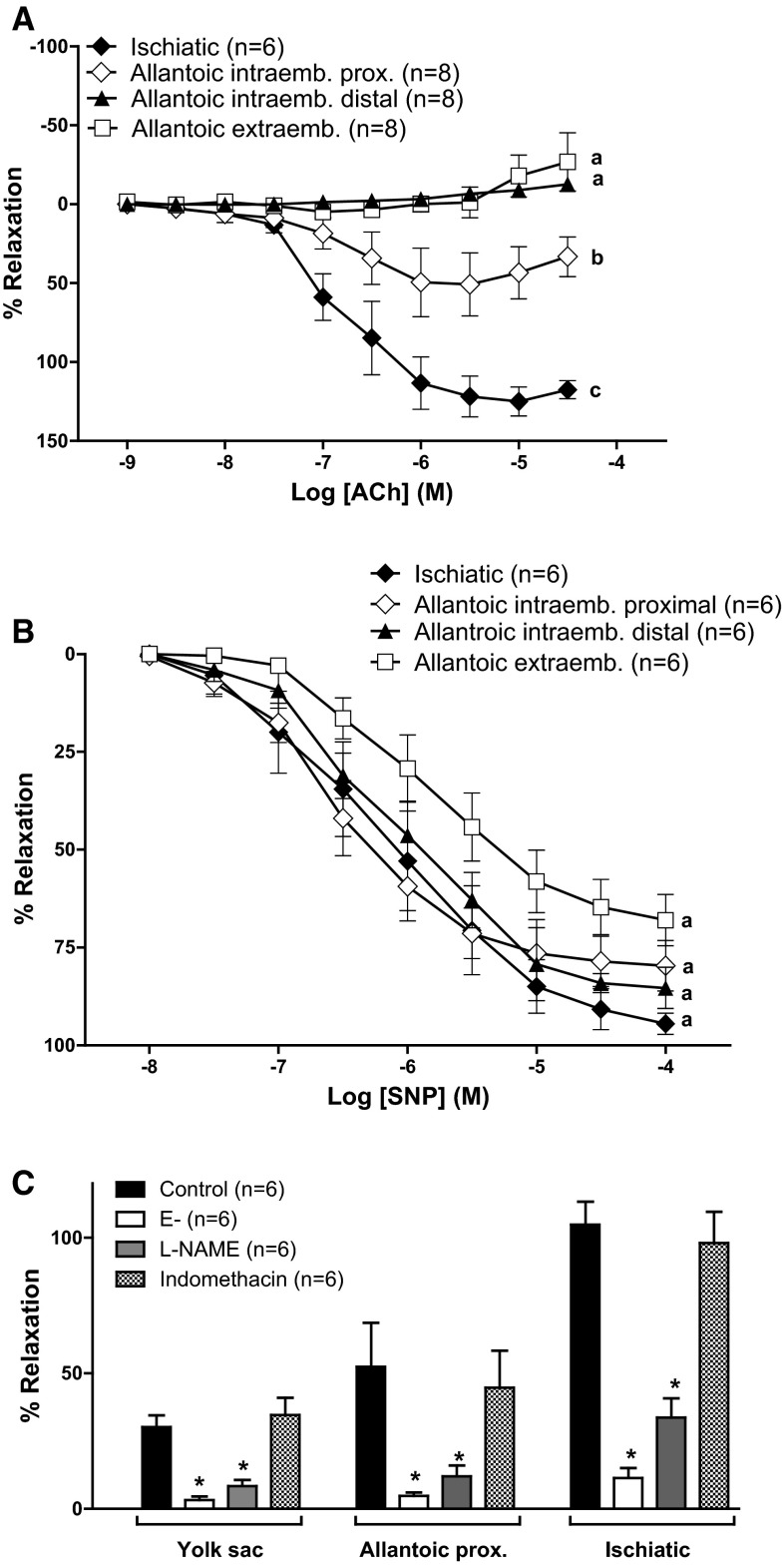
Segmental differences in the responsiveness of the allantoic artery to acetylcholine (ACh). **a** Relaxant effects of acetylcholine (ACh) in ischiatic, allantoic intraembryonic (proximal and distal) and allantoic extraembryonic arteries from 19-day chicken embryos. Vessels were pre-contracted with KCI (62.5 mM). Values (mean ± SE) without a *common letter* have a significantly different *E*_max_ (*p* < 0.05). **b** Effects of endothelium removal (E-), the NO synthase inhibitor L-NAME (0.1 mM) and the cyclooxygenase inhibitor indomethacin (10 µM) in the maximal relaxation evoked by ACh. *p < 0.05 vs. control

To investigate whether the lack of ACh-induced relaxation was a specific property of the extraembryonic part of the allantoic artery, we analyzed the response to the agonist in intraembryonic segments of the artery as well as in rings of the ischiatic artery. As shown in Fig. 7a, ACh-induced relaxation was present in the ischiatic and in the very proximal part of the allantoic artery but not in the distal intraembryonic or the extraembryonic part of the vessel. In contrast, no segmental differences were observed in the relaxation evoked by SNP (Fig. 7b).

Finally, we performed a further characterization of the relaxation induced by ACh in the ischiatic artery, the proximal intraembryonic part of the allantoic artery and the YS artery. As shown in Fig. 7c, ACh-induced relaxation was abolished by mechanical removal of the endothelium whereas SNP- and forskolin-induced relaxations were not affected by this (data not shown). The relaxant efficacy of ACh was impaired by the presence of the NO synthase inhibitor L-NAME (0.1 mM) but was not affected by the presence of the cyclooxygenase inhibitor indomethacin (10 μM, Fig. 7c).

### Developmental changes in allantoic and yolk sac arteries

**Fig. 8:**
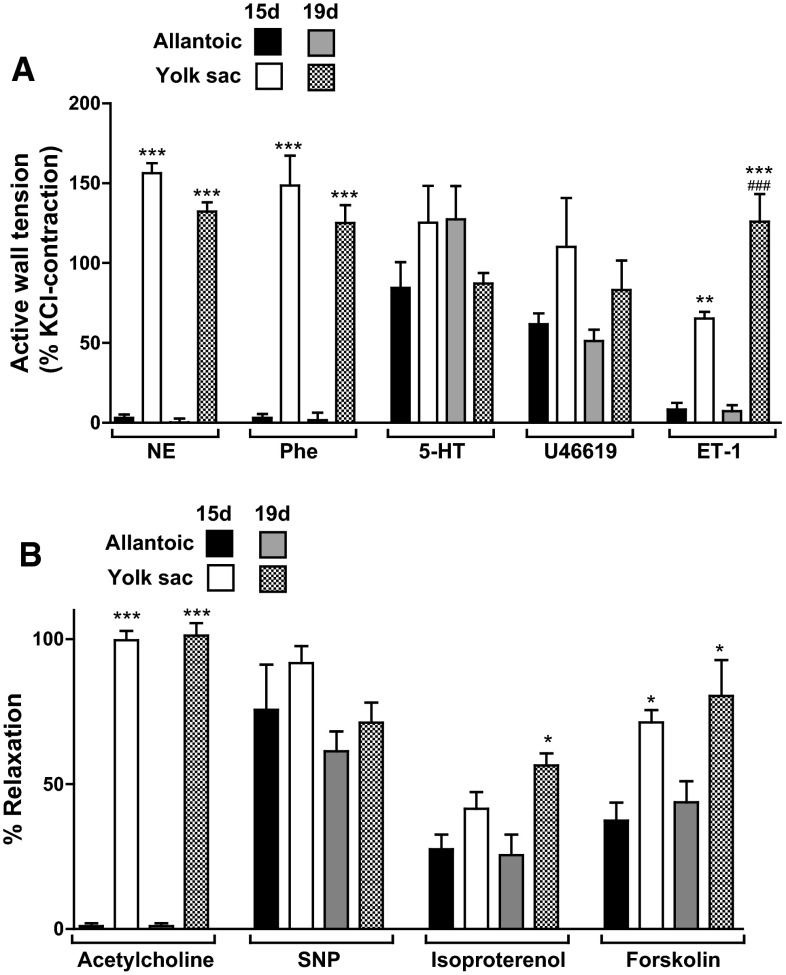
Developmental changes in the response of extraembryonic arteries (allantoic and yolk sac) from 15- and 19-day chicken embryos to contractile (**a**) and relaxant (**b**) agents. Relaxation to acetylcholine was studied in vessels pre-contracted with 5-HT (1 µM), whereas relaxation to sodium nitroprusside (SNP), isoproterenol and forskolin was studied in vessels pre-contracted with KCI (62.5 mM). Each *bar* represents the mean + SE of 4–10 embryos. *, **, ***p < 0.05, 0.01, 0.001 for difference (yolk sac vs. allantoic at the same age) in observed or apparent *E*_max_. ^###^ *p* < 0.001 for difference (15 vs. 19-days in the same vessel) in apparent *E*_max_

To investigate whether the lack of α-adrenergic-mediated contraction and ACh-induced relaxation was a specific property of the E19 allantoic artery, we analyzed the reactivity of E15 vessels. As shown in Fig. 8, the younger allantoic arteries neither responded to NE, phenylephrine nor ACh. In addition, no developmental changes were observed in the responsiveness of the allantoic arteries to any of the vasoactive agents tested (Fig. 8). ET-1 evoked a higher contraction in the E19 than in the E15 YS artery (Fig. 8a), but the responsiveness of the YS arteries to the other vasoactive agents did not change with incubation age (Fig. 8).

## Discussion

The present study demonstrates a different pattern of reactivity in the arteries perfusing the gas exchange and the nutritional organ (CAM and YS, respectively) of the late chicken embryo. In contrast to the YS artery, the allantoic artery did not show, in its extraembryonic part, catecholamine-containing nerves, α-adrenergic-mediated contraction or ACh-induced relaxation. Interestingly, periarterial nerves, α-adrenergic contraction and ACh-induced relaxation were present in the upper intraembryonic segment of the allantoic artery and decreased up to disappear along the trajectory of the vessel. β-Adrenergic relaxation was present in YS and allantoic arteries but it was significantly higher in the former vessel. ET-1 evoked a more efficacious contraction in the YS than in the allantoic artery, whereas the contractions evoked by 5-HT and the thromboxane A_2_ mimetic U46619 did not significantly differ between the two arteries. The relaxation evoked by the NO donor SNP did not significantly differ between the two artery types, whereas the adenylate cyclase activator forskolin induced a higher relaxation in the YS than in the allantoic arteries.

### Adrenergic responsiveness

Catecholamines play a key role in the prenatal cardiovascular response to stress and in several of the adaptations that characterize the transition from the pre- to the neonatal period (Mulder et al. 2000, 2001, 2002). In the mammalian fetus, the cardiovascular responses to acute hypoxia include a redistribution of the cardiac output away from the periphery towards high priority organs such as the heart, brain, and adrenal gland (Llanos et al. 2003). Similarly, in the chicken embryo acute hypoxia caused a redistribution of the cardiac output in favor of heart, brain and CAM at the expense of intestine, YS, liver, and carcass (Mulder et al. 1998). A component of this protective redistribution is mediated by increased release of catecholamines (Mulder et al. 2000, 2001, 2002), whose effects would be modulated by the different responsiveness of the vascular beds. Accordingly, we demonstrated that the allantoic arteries did not show α-adrenergic-mediated contraction but showed β-adrenergic-mediated relaxation. A similar pattern of adrenergic responsiveness has been reported in the small arteries of the CAM (Lindgren et al. 2010, 2011). In contrast, α-adrenergic contraction and β-adrenergic relaxation were present in the YS artery. Therefore, our results show a correlation between the distribution of cardiac output during hypoxia (i.e., increased to the CAM and decreased to the YS) and the local vascular responses to adrenergic stimulation. In addition, the extraembryonic part of the allantoic artery did not respond to EFS and did not show catecholamine-containing nerves. This implies that only humoral catecholamines can participate in the control of extraembryonic allantoic artery tone. The lack of contraction elicited by NE and phenylephrine in this vessel could be explained by low α-adrenoceptor density in the vessel and/or inefficient coupling between the adrenoceptors and the contractile machinery, although this remains to be determined.

In contrast to NE and phenylephrine, the α_2_-adrenoceptor agonist oxymetazoline elicited some contraction in the extraembryonic allantoic artery. It is unlikely that this weak contraction is due to a α_2_ effect because NE, which stimulates α_1_ and α_2_ receptors, had no effect in the extraembryonic part of the allantoic artery. Interestingly, it has been demonstrated that oxymetazoline is a potent agonist at several receptors of the 5-HT receptor family (Schoeffter and Hoyer 1991). We observed that allantoic arteries are responsive to 5-HT in a similar manner than YS arteries. Moreover, an important part of the contractile effect of oxymetazoline in the human umbilical artery is mediated through stimulation of 5-HT receptors (Bodelsson and Stjernquist 1995).

### Lack of ACh-induced relaxation in the extraembryonic allantoic arteries

ACh has been widely used in numerous vascular beds to stimulate endothelium-dependent relaxation (Furchgott and Zawadzki 1980; González-Luis et al. 2007; Ignarro 2002; Donald et al. 2015). Several endothelium-derived relaxing factors have been found, including nitric oxide (NO), prostaglandins, carbon monoxide and a yet unidentified factor called endothelium-derived hyperpolarizing factor (EDHF) (Busse et al. 2002; Baragatti et al. 2007; Donald et al. 2015). The contribution of each of these factors to endothelium-dependent relaxation varies across vascular beds and also with the physiological or pharmacological stimuli used to stimulate the endothelium (Busse et al. 2002). In the chicken embryo, ACh induced an endothelium-dependent and, at least partially, NO-mediated relaxation of the ductus arteriosus (Agren et al. 2007; Schuurman and Villamor 2010), the pulmonary (Villamor et al. 2002), the femoral (Villamor et al. 2002; le Noble et al. 2000), the mesenteric (Moonen and Villamor 2011) and the carotid arteries (le Noble et al. 2000). In contrast, ACh did not induce relaxation but contraction in the small arteries of the CAM (Lindgren et al. 2010). In the present study, we observed that ACh elicited an endothelium-dependent, NO-mediated relaxation in the YS artery and in the proximal (intraembryonic) part of the allantoic artery but not in the extraembryonic part of the vessel. In contrast, the NO donor SNP relaxed YS and allantoic arteries, indicating that the cGMP/sGC pathway of relaxation is functional in both vessels.

Besides ACh, the endothelial production of NO is stimulated by other substances, such as bradykinin, ATP, or the calcium ionophore A23187 (Ignarro 2002; Donald et al. 2015). Moreover, the endothelial cells themselves act as mechanoreceptors; and in cases of peripheral alterations of blood flow and viscosity, they generate signals for the production of NO (Kotsovolis and Kallaras 2010). When we tested ATP and A23187, we observed that they did not relax either allantoic or YS arteries. Therefore, these substances appear to be ineffective as relaxant agents in the chicken umbilical arteries. With our experimental setup, the effects of flow as endothelium-dependent vasodilator stimulus could not unfortunately be studied.

There are four principle causes of diminished NO bioactivity: decreased expression and/or activity of the endothelial NO synthase (eNOS) enzyme, eNOS uncoupling, enhanced breakdown or scavenging of NO and impaired transmission of NO-mediated signaling events (failure of the effector mechanisms) (Braam and Verhaar 2007). As mentioned above, the effector mechanisms do not appear to be affected in the allantoic arteries because they relax to a NO donor. The putative role of the other causes in the lack of responsiveness to ACh of extraembryonic allantoic arteries warrants further investigation.

As mentioned above, in some vessels the endothelium-dependent relaxation elicited by ACh is mediated by the release of EDHF. The identity of EDHF remains uncertain, but it is accepted that its action involves an increase in K^+^ conductance and can therefore be inhibited by abolishing the electrochemical gradient for K^+^ ions (Busse et al. 2002). Therefore, the lack of relaxation to ACh in the extraembryonic allantoic arteries might be due to the fact that the vessels were pre-contracted with a solution containing 62.5 mM K^+^. However, when 5-HT was used as pre-contractile agent, ACh-induced relaxation was also absent in the extraembryonic allantoic arteries, whereas it augmented significantly in the ischiatic, the YS and the intraembryonic allantoic arteries. These data suggest that EDHF could be involved in ACh-induced relaxation in the latter vessels but not in the extraembryonic allantoic arteries. In previous studies, we have shown the possible contribution of EDHF to ACh-induced relaxation in chicken embryo mesenteric arteries (Moonen and Villamor 2011) and ductus arteriosus (Agren et al. 2008).

### Cyclic nucleotide-mediated relaxation of chicken umbilical arteries

Cyclic nucleotides (cAMP and cGMP) are the main second messengers linked to vasodilatation (White et al. 2000). Activation of soluble guanylate cyclase and generation of cGMP is the main signal-transducing event of the l-arginine-NO pathway, whereas cAMP is the main intracellular second messenger of β-adrenergic- and prostanoid-mediated vascular relaxation (White et al. 2000). We observed that chicken allantoic and YS arteries were relaxed by SNP and forskolin, indicating that cGMP- and cAMP-mediated pathways of relaxation are functional in the chicken umbilical circulation.

As already mentioned, since extraembryonic allantoic arteries are not innervated, the low resistance of the allantoic circulation to blood flow is conferred by either humoral vasoactive agents or anatomical mechanisms. If low resistance to blood flow is brought about by the activities of vasoactive substances, then vasodilator mechanisms should predominate (Carter and Myatt 1995; Kingdom et al. 1994). However, an inappropriate or excessive relaxant response to relaxant agents may increase umbilical blood flow to the CAM and therefore shunt the blood away from the embryo body. Interestingly, we observed that the relaxant efficacy of forskolin was lower in allantoic than in YS arteries. Moreover, when compared with chicken embryo systemic and pulmonary arteries (Agren et al. 2008; Moonen and Villamor 2011; Villamor et al. 2002), allantoic arteries show lower sensitivity to forskolin and SNP. This low responsiveness to vasoactive agents suggests that the allantoic circulation might be in large part a passive circuit in which flow rate is determined by the mean effective perfusion pressure.

### Developmental changes in allantoic and yolk sac arteries

During prenatal life, endothelial and vascular smooth muscle cells play a key role in blood vessel morphogenesis and exhibit high rates of proliferation, migration, and production of extracellular matrix (Owens et al. 2004; Rzucidlo et al. 2007). These processes occur while the new forming vessels are simultaneously acquiring the capacity to regulate vascular tone and undergoing developmental changes in the contractile apparatus, the density of receptors and the signal transduction pathways leading to contraction/relaxation (Moonen and Villamor 2011). When reactivity is studied in prenatal vessels, it prevails frequently the assumption that full maturity is only achieved during postnatal life. Obviously, this is not the case of umbilical vessels. By days 14–15 of incubation the CAM capillary volume reaches a maximum and around the end of day 19, the CAM degenerates when lung ventilation is initiated during internal pipping (Ackerman and Rahn 1981). Therefore, our findings in the E19 allantoic artery could have been influenced by the fact that the vessel was close to the end of its functional life. Nevertheless, we observed that neither α-adrenergic-mediated contraction nor ACh-induced relaxation were present in the E15 allantoic arteries. Moreover, we did not observe any other incubation age-related changes in the responsiveness of allantoic arteries. In contrast, the YS arteries showed an increase with age in the responsiveness to ET-1. Interestingly, a similar age-related increase in ET-1-induced contraction can be observed in small mesenteric arteries, which also originate from the cranial mesenteric artery (Moonen and Villamor 2011). However, other changes observed in the mesenteric arteries, such as age-related increase in U46619-mediated contraction, as well as ACh- and forskolin-induced relaxation (Moonen and Villamor 2011), were not observed in the YS arteries. This suggests subtle differences in the pattern of maturation of the vessels supplying the prenatal and the postnatal nutritional organs.

### Comparison between chicken and mammalian umbilical arteries

From the two types of arteries, which are present in the chicken embryo umbilical system, the allantoic arteries are the ones with more in common with mammalian umbilical arteries. Lack of relaxation to ACh, lack of innervation, and low responsiveness to α-adrenoceptor agonists are also features of human (Hollingsworth 1974; Kalsner 1989; Clyman et al. 1975; Thornburg and Louey 2013; Bodelsson and Stjernquist 1995), mouse (Kusinski et al. 2009) and lamb (Dyer 1970; Arens et al. 1998) umbilical arteries. In contrast, ET-1 and U46619, which are potent stimulants of human and mouse umbilical arteries with pEC_50_ in the nanomolar range (Bodelsson and Stjernquist 1993; Bogoni et al. 1996; Kusinski et al. 2009), showed a very low potency in the chicken umbilical arteries, particularly in the allantoic artery. Also, 5-HT appears to be a more potent vasoconstrictor in the human (Tiritilli 2000; Santos-Silva et al. 2009) and lamb (Arens et al. 1998) than in the chicken umbilical arteries.

### Concluding remarks

In the present study, we confirmed our hypothesis of a different pattern of reactivity in the arteries perfusing the CAM and the YS membrane. Periarterial innervation, responsiveness to α-adrenoceptor agonists, and endothelium-dependent relaxation mediated by ACh were only present in the vessels supplying the nutritional membrane (i.e., the YS arteries) and in the upper part of the vessels supplying the respiratory membrane (i.e., the allantoic arteries). This first functional characterization of the two different vessels which form the chicken embryo umbilical arterial system will serve as the starting point of further investigation to explain the disparate and common responses. In addition, the segmental differences in the allantoic artery make this vessel an attractive model to study the mechanism regulating the development of neural and endothelial control of vascular tone.

## References

[CR1] Ackerman RA, Rahn H (1981). In vivo O_2_ and water vapor permeability of the hen’s eggshell during early development. Resp Physiol.

[CR2] Agren P, Cogolludo AL, Kessels CG, Perez Vizcaino F, De Mey JG, Blanco CE, Villamor E (2007). Ontogeny of chicken ductus arteriosus response to oxygen and vasoconstrictors. Am J Physiol Regul Integr Comp Physiol.

[CR3] Agren P, van der Sterren S, Cogolludo AL, Frazziano G, de Mey JG, Blanco CE, Villamor E (2008). Developmental changes in endothelium-dependent relaxation of the chicken ductus arteriosus. J Physiol Pharmacol.

[CR4] Agren P, van der Sterren S, Cogolludo AL, Blanco CE, Villamor E (2009). Developmental changes in the effects of prostaglandin E2 in the chicken ductus arteriosus. J Comp Physiol B.

[CR5] Arens Y, Chapados RA, Cox BE, Kamm KE, Rosenfeld CR (1998). Differential development of umbilical and systemic arteries II. Contractile proteins. Am J Physiol.

[CR6] Baragatti B, Brizzi F, Barogi S, Laubach VE, Sodini D, Shesely EG, Regan RF, Coceani F (2007). Interactions between NO, CO and an endothelium-derived hyperpolarizing factor (EDHF) in maintaining patency of the ductus arteriosus in the mouse. Br J Pharmacol.

[CR7] Bodelsson G, Stjernquist M (1993). Characterization of endothelin receptors and localization of 125I-endothelin-1 binding sites in human umbilical artery. Eur J Pharmacol.

[CR8] Bodelsson G, Stjernquist M (1995). Characterization of contractile adrenoceptors in the human umbilical artery. Eur J Pharmacol.

[CR9] Bogoni G, Rizzi A, Calo G, Campobasso C, D’Orleans-Juste P, Regoli D (1996). Characterization of endothelin receptors in the human umbilical artery and vein. Br J Pharmacol.

[CR10] Braam B, Verhaar MC (2007). Understanding eNOS for pharmacological modulation of endothelial function: a translational view. Curr Pharm Design.

[CR11] Busse R, Edwards G, Feletou M, Fleming I, Vanhoutte PM, Weston AH (2002). EDHF: bringing the concepts together. Trends Pharmacol Sci.

[CR12] Carter AM, Myatt L (1995). Control of placental blood flow: workshop report. Reprod Fertil Dev.

[CR13] Clyman RI, Sandler JA, Manganiello VC, Vaughan M (1975). Guanosine 3′,5′-monophosphate and adenosine 3′,5′-monophosphate content of human umbilical artery. J Clin Invest.

[CR14] Cogolludo AL, Moral-Sanz J, van der Sterren S, Frazziano G, van Cleef AN, Menendez C, Zoer B, Moreno E, Roman A, Perez-Vizcaino F, Villamor E (2009). Maturation of O_2_ sensing and signaling in the chicken ductus arteriosus. Am J Physiol Lung Cell Mol Physiol.

[CR15] Donald JA, Forgan LG, Cameron MS (2015). The evolution of nitric oxide signalling in vertebrate blood vessels. J Comp Physiol B.

[CR16] Dyer DC (1970). The pharmacology of isolated sheep umbilical cord blood vessels. J Pharmacol Exp Ther.

[CR17] Dzialowski EM, Sirsat T, van der Sterren S, Villamor E (2011). Prenatal cardiovascular shunts in amniotic vertebrates. Resp Physiol Neurobiol.

[CR18] Flinsenberg TW, van der Sterren S, van Cleef AN, Schuurman MJ, Agren P, Villamor E (2010). Effects of sex and estrogen on chicken ductus arteriosus reactivity. Am J Physiol Regul Integr Comp Physiol.

[CR19] Furchgott RF, Zawadzki JV (1980). The obligatory role of endothelial cells in the relaxation of arterial smooth muscle by acetylcholine. Nature.

[CR20] González-Luis G, Fletcher AJW, Moreno L, Perez-Vizcaino F, Blanco CE, Villamor E (2007). Nitric oxide-mediated nonadrenergic noncholinergic relaxation of piglet pulmonary arteries decreases with postnatal age. J Physio Pharmacol.

[CR21] Hollingsworth M (1974). Electrical stimulation and drug responses of isolated human umbilical blood vessels. Eur J Pharmacol.

[CR22] Ignarro LJ (2002). Nitric oxide as a unique signaling molecule in the vascular system: a historical overview. J Physiol Pharmacol.

[CR23] Jones CJ, Jauniaux E (1995). Ultrastructure of the materno-embryonic interface in the first trimester of pregnancy. Micron.

[CR24] Kalsner S (1989). Cholinergic constriction in the general circulation and its role in coronary artery spasm. Circ Res.

[CR25] Kingdom JC, Macara LM, Whittle MJ (1994). Fetoplacental circulation in health and disease. Arch Dis Child.

[CR26] Kotsovolis G, Kallaras K (2010). The role of endothelium and endogenous vasoactive substances in sepsis. Hippokratia.

[CR27] Kusinski LC, Baker PN, Sibley CP, Wareing M (2009). In vitro assessment of mouse uterine and fetoplacental vascular function. Reprod Sci.

[CR28] le Noble FA, Ruijtenbeek K, Gommers S, de Mey JG, Blanco CE (2000). Contractile and relaxing reactivity in carotid and femoral arteries of chicken embryos. Am J Physiol Heart Circ Physiol.

[CR29] Levinsohn EM, Packard DS, West EM, Hootnick DR (1984). Arterial anatomy of chicken embryo and hatchling. Am J Anat.

[CR30] Lindgren I, Zoer B, Altimiras J, Villamor E (2010). Reactivity of chicken chorioallantoic arteries, avian homologue of human fetoplacental arteries. J Physiol Pharmacol.

[CR31] Lindgren I, Crossley D, Villamor E, Altimiras J (2011). Hypotension in the chronically hypoxic chicken embryo is related to the beta-adrenergic response of chorioallantoic and femoral arteries and not to bradycardia. Am J Physiol Regul Integr Comp Physiol.

[CR32] Llanos AJ, Riquelme RA, Sanhueza EM, Hanson MA, Blanco CE, Parer JT, Herrera EA, Pulgar VM, Reyes RV, Cabello G, Giussani DA (2003). The fetal llama versus the fetal sheep: different strategies to withstand hypoxia. High Alt Med Biol.

[CR33] Moonen RM, Villamor E (2011). Developmental changes in mesenteric artery reactivity in embryonic and newly hatched chicks. J Comp Physiol B.

[CR34] Moonen RM, Kessels CG, Zimmermann LJ, Villamor E (2012). Mesenteric artery reactivity and small intestine morphology in a chicken model of hypoxia-induced fetal growth restriction. J Physiol Pharmacol.

[CR35] Mulder AL, van Golde JC, Prinzen FW, Blanco CE (1998). Cardiac output distribution in response to hypoxia in the chick embryo in the second half of the incubation time. J Physiol.

[CR36] Mulder AL, Golde JM, Goor AA, Giussani DA, Blanco CE (2000). Developmental changes in plasma catecholamine concentrations during normoxia and acute hypoxia in the chick embryo. J Physiol.

[CR37] Mulder AL, van Goor CA, Giussani DA, Blanco CE (2001). Alpha-adrenergic contribution to the cardiovascular response to acute hypoxemia in the chick embryo. Am J Physiol Regul Integr Comp Physiol.

[CR38] Mulder AL, Miedema A, De Mey JG, Giussani DA, Blanco CE (2002). Sympathetic control of the cardiovascular response to acute hypoxemia in the chick embryo. Am J Physiol Regul Integr Comp Physiol.

[CR39] Owens GK, Kumar MS, Wamhoff BR (2004). Molecular regulation of vascular smooth muscle cell differentiation in development and disease. Physiol Rev.

[CR40] Renfree MB, Suzuki S, Kaneko-Ishino T (2013). The origin and evolution of genomic imprinting and viviparity in mammals. Philos Trans R Soc Lond Ser B Biol Sci.

[CR41] Rouwet EV, De Mey JG, Slaaf DW, Heineman E, Ramsay G, Le Noble FA (2000). Development of vasomotor responses in fetal mesenteric arteries. Am J Physiol Heart Circ Physiol.

[CR42] Ruijtenbeek K, Kessels CG, Villamor E, Blanco CE, De Mey JG (2002). Direct effects of acute hypoxia on the reactivity of peripheral arteries of the chicken embryo. Am J Physiol Regul Integr Comp Physio.

[CR43] Rzucidlo EM, Martin KA, Powell RJ (2007). Regulation of vascular smooth muscle cell differentiation. J Vasc Surg.

[CR44] Santos-Silva AJ, Cairrao E, Marques B, Verde I (2009). Regulation of human umbilical artery contractility by different serotonin and histamine receptors. Reprod Sci.

[CR45] Schoeffter P, Hoyer D (1991). Interaction of the alpha-adrenoceptor agonist oxymetazoline with serotonin 5-HT1A, 5-HT1B, 5-HT1C and 5-HT1D receptors. Eur J Pharmacol.

[CR46] Schuurman MJ, Villamor E (2010). Endothelium-dependent contraction induced by acetylcholine in the chicken ductus arteriosus involves cyclooxygenase-1 activation and TP receptor stimulation. Comp Biochem Physiol A Mol Integr Physiol.

[CR47] Speake BK, Murray AM, Noble RC (1998). Transport and transformations of yolk lipids during development of the avian embryo. Prog Lipid Res.

[CR48] Thornburg KL, Louey S (2013). Uteroplacental circulation and fetal vascular function and development. Curr Vasc Pharmacol.

[CR49] Tiritilli A (2000). 5-hydroxytryptamine induces vasoconstriction of the human umbilical artery: effects of hypoxia and nicorandil. Gynecol Obstet Invest.

[CR50] van der Sterren S, Kleikers P, Zimmermann LJ, Villamor E (2011). Vasoactivity of the gasotransmitters hydrogen sulfide and carbon monoxide in the chicken ductus arteriosus. Am J Physiol Regul Integr Comp Physiol.

[CR51] van Golde J, Mulder T, Straaten HV, Blanco CE (1996). The chorioallantoic artery blood flow of the chick embryo from stage 34 to 43. Pediatr Res.

[CR52] Villamor E, Ruijtenbeek K, Pulgar V, De Mey JG, Blanco CE (2002). Vascular reactivity in intrapulmonary arteries of chicken embryos during transition to ex ovo life. Am J Physiol Regul Integr Comp Physiol.

[CR53] Villamor E, Kessels CG, Ruijtenbeek K, van Suylen RJ, Belik J, de Mey JG, Blanco CE (2004). Chronic in ovo hypoxia decreases pulmonary arterial contractile reactivity and induces biventricular cardiac enlargement in the chicken embryo. Am J Physiol Regul Integr Comp Physiol.

[CR54] White PT (1974). Experimental studies on the circulatory system of the late chick embryo. J Exp Biol.

[CR55] White RE, Kryman JP, El-Mowafy AM, Han G, Carrier GO (2000). cAMP-dependent vasodilators cross-activate the cGMP-dependent protein kinase to stimulate BK(Ca) channel activity in coronary artery smooth muscle cells. Circ Res.

[CR56] Yadgary L, Kedar O, Adepeju O, Uni Z (2013). Changes in yolk sac membrane absorptive area and fat digestion during chick embryonic development. Poult Sci.

[CR57] Zoer B, Blanco CE, Villamor E (2010). Role of Rho-kinase in mediating contraction of chicken embryo femoral arteries. J Comp Biol B.

[CR58] Zoer B, Cogolludo AL, Perez-Vizcaino F, De Mey JG, Blanco CE, Villamor E (2010). Hypoxia sensing in the fetal chicken femoral artery is mediated by the mitochondrial electron transport chain. Am J Physiol Regul Integr Comp Physiol.

